# *Mycobacterium marinum* infection in fish and man: epidemiology, pathophysiology and management; a review

**DOI:** 10.1080/01652176.2018.1447171

**Published:** 2018-03-21

**Authors:** Emad Hashish, Abdallah Merwad, Shimaa Elgaml, Ali Amer, Huda Kamal, Ahmed Elsadek, Ayman Marei, Mahmoud Sitohy

**Affiliations:** a Department of Clinical Pathology, Faculty of Veterinary Medicine, Zagazig University, Egypt; b Department of Zoonoses, Faculty of Veterinary Medicine, Zagazig University, Egypt; c Tuberculosis Unit, Animal Health Research Institute (AHRI), Giza, Egypt; d Department of Meat Hygiene, National Research Center (NRC), Zagazig, Egypt; e Immunology Research Lab, Immunology Division, Department of Microbiology and Immunology, Faculty of Medicine, Zagazig University, Egypt; f Department of Biochemistry, Faculty of Agriculture, Zagazig University, Egypt

**Keywords:** Fish, *Mycobacterium marinum*, epidemiology, pathophysiology, public health, virulence factors

## Abstract

*Mycobacterium marinum* is an opportunistic pathogen inducing infection in fresh and marine water fish. This pathogen causes necrotizing granuloma like tuberculosis, morbidity and mortality in fish. The cell wall-associated lipid phthiocerol dimycocerosates, phenolic glycolipids and ESAT-6 secretion system 1 (ESX-1) are the conserved virulence determinant of the organism. Human infections with *Mycobacterium marinum* hypothetically are classified into four clinical categories (type I–type IV) and have been associated with the exposure of damaged skin to polluted water from fish pools or contacting objects contaminated with infected fish. Fish mycobacteriosis is clinically manifested and characterized in man by purple painless nodules, liable to develop into superficial crusting ulceration with scar formation. Early laboratory diagnosis of *M. marinum* including histopathology, culture and PCR is essential and critical as the clinical response to antibiotics requires months to be attained. The pathogenicity and virulence determinants of *M. marinum* need to be thoroughly and comprehensively investigated *and* understood. In spite of accumulating information on this pathogen, the different relevant data should be compared, connected and globally compiled. This article is reviewing the epidemiology, virulence factors, diagnosis and disease management in fish while casting light on the potential associated public health hazards.

## 1. Introduction

Fish mycobacteriosis is a chronic progressive disease caused by ubiquitous acid-fast bacilli, identified as nontubercolous mycobacteria (NTM) (Novotny et al. 2004). NTM could be classified into slowly and rapidly growing mycobacteria, where *Mycobacterium marinum* is affiliated to the first class. *Mycobacterium marinum*, *M. fortuitum* and *M. chelonae* are among the most identified NTM mycobacterial species associated with fish mycobacteriosis (Novotny et al. 2004). Piscine mycobacteriosis is a common disease of marine, brackish and freshwater fish (Decostere et al. 2004) infecting more than 200 species of freshwater and marine fish in a vast region extending from the subarctic zone to the tropical one (Zanoni et al. 2008; Gauthier and Rhodes 2009; Jacobs et al. 2009; Ackleh et al. 2015). This disease also infects tropical aquarium fish (Noga 2010) and is considered a major cause of morbidity and mortality in free-living fish (Chang and Whipps 2015).

*Mycobacterium marinum* is an environmental, aerobic waterborne bacterium, belonging to photochromogenic Group I non-tuberculous *Mycobacteria* according to Runyon's classification. It has a relatively short generation time amounting to 4–6 h as compared to 20 h in the case of *M. tuberculosis*, and optimally grows at 30 °C but hardly at 37 °C (Clark and Shepard 1963). *M. marinum* is one of the most common atypical *Mycobacteria* that cause human opportunistic infection (Rallis and Koumantaki-Mathioudaki 2007). It is considered as the most important fish pathogen, associated with multiple symptoms, e.g. uncoordinated swimming, abdominal swelling, loss of weight, skin ulceration, white nodule formation as granuloma in liver, kidney, spleen in both fresh and marine water fish (Ferguson 2006; El Amrani et al. 2010).

The transmission of *M. marinum* between fishes is poorly understood. The primary route of infection is the oral one via consumption of infected dead fish, contact with affected fish skin or through gills (El Amrani et al. 2010). *M. marinum* outbreaks were reported in striped bass in USA (Gauthier et al. 2008), in sturgeon fish in China (Zhang et al. 2015) and in F1 captive-bred Australian lung fish (Strike et al. 2016). Fish mycobacteriosis has become an important threat to aquatic industry coinciding with the rapid development of sturgeon's culture in China (Li et al. 2009). Mixed mycobacterial infections have an economic impact in sturgeon in China, being associated with more than 50% mortality during 2009–2010 (Zhang et al. 2015).

*Mycobacterium marinum* is posing a zoonotic concern, since it produces granulomatous lesions in human skin and deep tissues (Lewis et al. 2003; Petrini 2006). Additionally, it causes cutaneous infections manifested by ulcers, skin nodules and nodular lymphangitis, when exposed to abrasions and lacerations in salt water or injuries associated with fish spines (Jernigan and Farr 2000). Human infection consists of nodular cutaneous lesions that could progress to tenosynovitis, arthritis and osteomyelitis (Aubry et al. 2002; Wongworawat et al. 2003). Humans may contract infection with a fish spine injury occurring at a fishing pond, in the fish processing industry or through contaminated pond water (Tsai et al. 2007).

Since the infection severity of *M. marinum* is very wide, ranging from spontaneous healing to deep long-lasting infection (Aubry et al. 2002), the identification of existent or emerging virulent strains of *M. marinum* is highly recommended for the control of this potential human health hazard. Many versatile genotyping methods are being developed for classifying *M. marinum* such as variable number of tandem repeat (van der Sar et al. 2004; Ucko and Colorni 2005). The ensuing results of such studies on *M. marinum* need to be compared, correlated, connected and globally compiled. In response to this need, this article is reviewing the epidemiology, virulence factors, diagnosis and disease management in fish while casting light on the potential associated public health hazards and focusing on the zoonotic significance of this pathogen and referring to the possible areas of insufficient studies.

## 2. Etiological agent and ecology

Piscine mycobacteriosis is caused by different NTM mycobacterial species. *M. marinum* was first reported in marine fish by Von Betegh (1910) and first isolated by Aronson (1926). *M. marinum* belongs to the Order Actinomycetales, Suborder Corynebacterineae and Family Mycobacteriaceae. It induces fish tank granuloma in humans, which was first described in 1951, as resulting from ‘*Mycobacterium balnei*’ (Nordén and Linell 1951). Linell and Norden (1954) recognized this pathogen after investigating eighty persons who used the same public swimming pool and were affected by granulomatous lesions in their skin. Later on the causative agent of piscine mycobacteriosis in man was confirmed as *M. marinum* (Swift and Cohen 1962).

This bacterium is pleomorphic, Gram-positive, acid-fast, aerobic, taking the shape of non-motile rods. The bacterium cell wall is specifically rich in mycolic acids, which are the major and specific lipid component in the cell wall envelope. They are 3-hydroxy long chain fatty acid (60–90 carbon atoms) (Gangadharam and Jenkins 1997) which are essential for the survival of members of the genus (Marrakchi et al. 2014). The genus includes approximately 75 species of close relations based on similarities in their sequences of 16S rRNA (Goodfellow and Magee 1998). Additionally, the cell wall envelope of *Mycobacterium* sp. comprises other lipids, e.g. trehalose 6,6-dimycolate (TDM), lipoarabinomannan (LAM), phthiocerol dimycocerosates (PDIMs), phenolic glycolipids (PGLs) (Draper 1991; Daffé and Draper 1997; Smith 2003; Onwueme et al. 2005). These groups of lipids are closely associated with the virulence of *Mycobacterium* (Brennan 2003; Smith 2003; Reed et al. 2004).

*M. marinum* is an ubiquitous waterborne bacterium with an optimal growth temperature around 30 °C, which causes the infection of bats, fish, mice and amphibians after its inoculation (Clark and Shepard 1963). Experimental infection of mice with *M. marinum* at a temperature below 30 °C developed pulmonary lesions, but not at 34 °C (Clark and Shepard 1963). *M. marinum* is prevalent all over the world in marine water, brackish water, and fresh water and naturally infects more than 150 species of fish, frog, freshwater eels and oyster (Beecham et al. 1991; Zeeli et al. 2003). In Africa, *M. marinum* has been isolated from normal humans’ skin and also from soil (Pattyn, 1984). It grows on Lowenstein–Jensen media at 30 °C within duration of 2–3 weeks (Vincent et al. 2003).

*M. marinum* can invade macrophages, preventing the fusion of phagosome-lysosome and replicating inside (El-Etr et al. 2001). *M. marinum* strains could be classified into two virulence groups based on their genetic diversity. Molecular variations were spotted among *M. marinum* strains isolated from different environments (Ang et al. 2000). Two clusters (I and II) of *M. marinum* could be defined with the use of amplified fragment length polymorphism. Cluster I of *M. marinum* was isolated from humans suffering from fish tank granuloma and with acute infection symptoms, while cluster II strains were originating from poikilothermic species and were predominantly associated with chronic infection (van der Sar et al. 2004). Hence, the detection of some genotypes of *M. marinum* in humans may suggest variable zoonotic potentials.

*M. marinum* strains infecting humans were isolated from swimming pools, wells, rivers and fish tanks. *Mycobacterium* spp. are opportunistic pathogens that residue in aquaria environment and water supplies (Yanong et al. 2010). This pathogenic organism produces a yellow pigment when subjected to light and uses a similar pathogenic mechanism for survival and replication inside the host macrophages, forming a granuloma during chronic infection (Barker et al. 1997; Tobin and Ramakrishnan 2008).

Clinical isolates of *M. marinum* from human and fish sources isolated from the same country were reported to have genetic variations (Ucko and Colorni 2005). It was cited that *M. marinum* is very closely related to *M. ulcerans*, and the two species are suggested to be in close relation to *M. tuberculosis* based on 99.3% similarity in 16S rRNA sequences (Roberts, 2001), DNA/DNA hybridization and comparative fatty acid profiles (Tønjum et al. 1998). Strains of *M. marinum* derived from fish reveal considerable differences in phenotypic, genetic and virulence properties (Sechi et al. 2002; Ucko et al. 2002). The comparative analysis of genomic sequences of *M. marinum* strains from fish and man will have beneficial implications, enabling the development of novel molecular markers for detection and facilitating the study of the epidemiology and evolution of the pathogen (Kurokawa et al. 2013).

## 3. Epidemiology in fish

*Mycobacterium* sp. have been identified as one of the most important micro-organisms causing morbidity and mortality in cultivated and wild fishes all over the world (Gauthier and Rhodes 2009). It has been recorded in fresh water and marine fish in the tropical and subarctic regions (Frerichs 1993; Diamant et al. 2000; Rhodes et al. 2004). The cultured water fish species including sea bass and striped bass (Hedrick et al. 1987), the *Oreochromis mossambicus* fish (Noga et al. 1990) and freshwater fishes belonging to the families Anabantidae, Characidae and Cyprinidae (Astrofsky et al. 2000) are considered especially susceptible to the infection with *Mycobacterium*.

The infection mostly occurs within 2 weeks after exposure to aquariums or after direct inoculation of the organism either from fish fins or bites. Moreover, the infection rates may have a wide range of 10%–100% (Francis-Floyd and Yanong 2002). Three species of *Mycobacterium* (*M. marinum*, *M. fortuitum* and *M. chelonae*) have been recorded to induce pathogenicity in fish (Novotny et al. 2004; Gauthier and Rhodes 2009). Water and related biofilms are the natural habitats of these three species (Pedley et al. 2004). The first species is slowly growing (Tønjum et al. 1998) while the other two are rapidly growing mycobacterium (Han et al. 2007).

*M. marinum* is the most common species isolated from various fishes and is recognized as a human pathogen (Gauthier et al. 2003; Jacobs et al. 2009). It causes episodes of infections in the cultured fish (dos Santos et al. 2002; Brocklebank et al. 2003; Aranaz et al. 2008), but it was also reported responsible for sporadic infections in rainbow and brown trout in Italy (Salogni et al. 2007) and was also isolated from shellfish (Beecham et al. 1991).

A case of *Mycobacterium marinum* infection was described in a farm of hybrid striped bass in Italy (Bozzetta et al. 2010). This species was considered as a potential source of infections for humans during sport activities and also a hazard risk for workers in the fish farm. The economic effect of mycobacteriosis in fish industry is still underestimated owing to the long incubation period, chronic nature and difficult diagnosis particularly at the early infection stage (Chinabut 1999). *M. marinum* was the most prevalent species (89.5%) causing sturgeon mycobacteriosis as evidenced by PCR-denaturing gradient gel electrophoresis and library sequencing of rpoB gene (Zhang et al. 2015).

Fish acquire the mycobacteriosis infection through the consumption of contaminated food and polluted water (Gauthier et al. 2003; Nenoff and Uhlemann 2006). Transovarian transmission was also recorded in viviparous fish (Frerichs 1993) and in live bearing fishes (Conroy 1966). The vertical transmission of mycobacteria was previously explained (Chinabut 1999), although this transmission route has not been documented in salmonids (Astrofsky et al. 2000). Zebra fish embryos were infected with *M. marinum* via bath exposure (Davis et al. 2002), while adult zebra fish were infected through the gut rather than gills via gavage and bath exposure (Harriff et al. 2007).

The digestive transmission of *M. marinum* was also suggested when the digestive tract was infected in 16.5% of the horse mackerel (Ortega et al. 2014) in spite of sporadic detection of granuloma in gills. Thereby, effective control measures of mycobacteriosis in fish farm should consider the gut as a natural route of exposure to infection with *M. marinum* (Harriff et al. 2007). Transmission of piscine mycobacteriosis occurs through ingestion of contaminated feed and cannibalism of infected fish (Chinabut et al. 1990; Grady et al. 1992).

In summary, the disease is transmitted through skin injuries and the external bacteria are included in the cutaneous transmission route (Post 1987). Aquatic vertebrates as frogs and turtles are sources for fish infection with mycobacteriosis. Also, snails are believed to be an important reservoir in aquarium fish (Post 1987). Additionally, water fleas were also reported as source of *M. marinum* infection when aquarium fish fed on water fleas harboring this pathogen (Grange et al. 1985). Similarly it has been reported that contaminated water fleas are responsible for producing characteristic granulomata of fish mycobacteriosis in Siamese fighting fish, when they were used as a source of live food for rearing this fish (Sodjit et al. 1993).

Prevalence of fish mycobacteriosis had been reported in previous studies (Gauthier et al. 2011). In a study from the Chesapeake Bay, *M. marinum* was isolated in only 3% (6 out of 192) examined spleen samples from striped bass *Morone saxatilis* (Rhodes et al. 2004). In another study, only one isolate of *M. marinum* was revealed from 106 kidney samples from fighting fish *Betta* spp after subculturing on Lowenstein–Jensen agar, Ziehl Neelsen acid-fast staining and 16 S rRNA sequencing (Najiah et al. 2011).

In a survey over the period 2002–2005 in Italy, the isolation percentages of *M. marinum* from positive ornamental fish were around 4.4% (Zanoni et al. 2008). A much higher percentage of 41.7% was isolated from gills, muscle and intestine of ornamental fish against a medium value of 19.3% from environmental samples of aquaria fish (Slany et al. 2014). This higher spread of *M. marinum* in these ornamental fishes was attributed to high density of fish, optimum water temperature of aquarium (close to 25 °C) and the frequent transfer of fish between the aquaria (Slany et al. 2014), the environmental factors which keep the aquarium most suitable for the spread of fish mycobacteriosis. Alternatively, host factors may play an additional role in the prevalence of piscine mycobacteriosis, based on the variable susceptibility of medaka and zebrafish for *M. marinum* (Broussard and Ennis 2007).

The initial dose of infecting *M. marinum* was advocated to be an influential factor, since low initial doses were associated with chronic disease, while high dose (>10^7^ CFU/g) were associated with acute and sub-acute infections (Prouty et al. 2003). The reported substantial genetic variation of *M. marinum* strains between fish and humans (Ucko and Colorni 2005) may also play a role in the nature of established infection. For example, Zebra fish developed acute infection when inoculated with *M. marinum* isolate of human origin, but chronic disease when inoculated with *M. marinum* isolate of fish origin (van der Sar et al. 2004).

## 4. Clinical signs in fish

*M. marinum* causes chronic, progressive disease of Zebra fish in a dose-dependent fashion. The induced tuberculosis in the zebra fish is similar to active human tuberculosis, where the predominant lesion at late stages from the infection are the presence of necrotizing granulomas, hallmark lesion of active tuberculosis with abundant bacteria in the necrotic areas (Swaim et al. 2006). Acute infection was characterized by uncontrolled development of the pathogen and death of all fish within 16 days, while chronic infections were marked by the formation of granuloma in different organs and longer survival in the range of 4–8 weeks (van der Sar et al. 2004).

*M. marinum*-induced tuberculosis in adult zebra fish; most of zebrafish died within 2 weeks of infection with 8970 bacteria, while infection with 5 CFU caused 44% mortality and infection with 60 CFU caused 83% mortality by 16 weeks. The infected fish showed a reduction in feed intake and weakened swimming for 1 week during the period prior to death. The infected fish either remained listless at the bottom or at the surface, constantly opening and closing their mouths to increase the gas exchange. The dying fish were often marked with external red lesions on the trunk ventral to the lateral line, an exophthalmia with ascites in close proximity to internal organs, where the former two signs are indicators for osmoregulatory stress and kidney failure (Swaim et al. 2006).

## 5. Pathogenesis and virulence determinants

Many virulence determinants and pathogenicity mechanisms are shared between *M. marinum* and *M. tuberculosis complex*. *M. marinum* is an opportunistic human pathogen causing a TB-like infection in the ectotherms, and also, can induce caseation granulomas in Zebra fish, similar to those composed in man (Tobin and Ramakrishnan 2008). The exact mechanism of the host's immune reaction against *M. marinum* still needs to be thoroughly investigated and explored. It has been established that *M. marinum* actively stimulates actin-polymerization within phagosomes by a complicated cellular mechanism enabling the organism to propel itself, by actin-based motility, into adjacent cells. Thus, *M. marinum* can escape into the cytoplasm of the infected macrophages in an RD1-dependent fashion, then spreading again from cell to cell, circumventing the host defense mechanisms and promoting permanent infection (Stamm et al. 2003). Nevertheless, the absence of macrophages during early infection with *M. marinum* was reportedly associated with higher bacterial burden indicating the macrophages control the infection at early stages and are not a niche for optimal growth (Clay et al. 2007).

*M. marinum v*irulence determinants causing granulomas are not fully identified. Many hosts like adult and embryonic zebra fish, animals and protozoa were utilized as models to study *M. marinum* pathogenesis and host-pathogen interactions (Davis et al. 2002). In a study on gold fish, the signature-tagged mutagenesis (STM) was utilized for characterization of *M. marinum* virulence genes needed for its *in-vivo* survival (Ruley et al. 2004), identifying 33 putative virulence genes, only 5 of which are homologues with *M. tuberculosis*. These 5 genes included pks genes, PPE family genes, and a transcriptional regulator with an AraC signature (Ruley et al. 2004).

Several *M. marinum* genes and pathways were found to have almost the same effects in different hosts. These conserved virulence determinants vary from secreted molecules to cell envelope biosynthesis and to cell physiology adaptation. An example of conserved virulence determinant of the organism is the cell wall-associated lipid PDIM, PGLs and ESAT-6 secretion system 1 (ESX-1) which if interrupted, attenuation of *M. marinum* in mammalian, fish, and protozoan cells will occur, referring to their significance for the pathogen in different hosts (Weerdenburg et al. 2015; Huang et al. 2016). In *M. tuberculosis*, PDIM and ESAT-6 have been concluded important for the infection as well (Sassetti and Rubin 2003), indicating that both genes are conserved across mycobacterial species. In addition to these genes, the mammalian cell entry 4 (mce4) and mce1 gene clusters could also be identified as general virulence factors of both *M. marinum* and *M. tuberculosis* (Sassetti and Rubin 2003; Senaratne et al. 2008), where *mce4* gene encodes a cholesterol import system providing energy to mycobacteria during infection (Pandey and Sassetti 2008).

Many common virulence determinants could be identified, other virulence genes had a very crucial effect in a narrow range of host cells and thus may be utilized as indicators for adaptation of bacterial host (Weerdenburg et al. 2015). An example of these host-related genes are *M. marinum* specific set of gene cluster that are homologues to ESX-1 cluster. Proteins of ESX-1 have a very definite role in host adaptation. Orthologues of these genes were recovered only in *M. marinum* or *M. liflandii* (Weerdenburg et al. 2015).

Many studies have firmly established a role for ESX-1 in virulence, both in *M. marinum* and in *M. tuberculosis*. This type VII secretion system, which is encoded by the *esx-1* gene cluster, is responsible for the secretion of numerous virulence factors (Sechi et al. 2002; Bottai and Brosch 2009; Weerdenburg et al. 2015). In contrast to *M. tuberculosis*, *M. marinum* has almost two identical copies of the ESX-1 substrates esxAB. The extra set appears to be specifically necessary for the infection of the fish cell line CLC, suggesting a host-specific ESX-1 function (Weerdenburg et al. 2015).

One of the features of *M. marinum* pathogenicity is its exclusive ability to access the macrophage cytosol and utilize host actin for motility (Stamm et al. 2003). This ability was not noticed in bacteria lacking ESX-1 system and thereby, growth attenuation was detected in different hosts (Cosma et al. 2004; Stoop et al. 2011; Kennedy et al. 2014). Recently, a novel protein secretion system named as ESX-5 gene, which was proven restricted *to M. tuberculosis* and *M. marinum* (Abdallah et al. 2006; Abdallah et al. 2009). ESX-5 is responsible for the transport of more than 100 proteins of PE and PPE families in *M. marinum* (Daleke et al. 2011). In another study, ESX-5 deficient *M. marinum* was attenuated in embryos of zebra fish indicating the importance of ESX-5 gene for *M. marinum* to establish and persist infections (Weerdenburg et al. 2012).

The pathogenicity of mycobacteria is associated with their capacity to export different virulence factors, for which mycobacteria own many protein secretion systems. SecA2 was identified in the pathogenic mycobacteria as a mycobacterial Sec translocation pathway involved in protein translocation across the inner membrane (Braunstein et al. 2003) to evade the innate and adaptive immune responses (Jensen et al. 2012; Watkins et al. 2012). The association between Sec A2 attenuation and the inability of mycobacteria to block the maturation of phagosome (Sullivan et al. 2012) confirms its essential role in developing pathogenicity. Identifying SecA2 substrates in *M. marinum* confirms that protein kinase G (PknG) can play an important role as a virulence effecter. Partial regaining of secA2 virulence following the overexpression of PknG confirms the role played by this protein in controlling the mycobacterial virulence and restoring blockage of phagosomal maturation (van der Woude et al. 2014).

## 6. Hematological changes in fish

The changes in the hematological parameters may indicate fish physiological responses against different environmental stresses and infections. *Tilapia* infected with *M. marinum* manifested an overall decrease in erythrocyte number and haematocrit with hypochromic microcytic anemia associated with a significant increase in circulating immature erythrocytes (Barham et al. 1980).

In another study, the hematological parameters exhibited nonsignificant alterations in the erythrocytic indices of Nile *Tilapia* inoculated with *M. marinum*, for eight days at 30 °C with only individual cases of hypochromic, microcytic anemia and wide variations in thrombocyte count. Leukocytosis occurred in fish after one day of infection as a result of neutrophilia and slight lymphocytosis, being the most characteristic feature of acute infection. After 3 days of infection, chronicity signs began to appear in the form of lymphocytosis and neutropenia, while after 14th and 35th days and up to the 45th day the monocytosis emerged and prevailed. Eosinophilia was only noticed in one fish at the 14th day after infection. Some other morphological abnormalities including immature cells, cytoplasmic vacuolisation in the monocytes, toxic cytoplasmic granulations in neutrophils and intensive cytoplasmic basophilia were recorded in infected fish at different times (Ranzani-Paiva et al. 2004).

## 7. Histopathological investigation in fish

Histopathological examinations mostly demonstrate a nonspecific inflammatory infiltration of epithelioid cells, lymphocytes and Langhan's giant cells without caseation. The early lesions commonly reveal a collection of polymorphonuclear cells surrounded with histiocytes. The histopathological examination might be significantly consolidated by the rapidly improving possibilities of vivo imaging, such as magnetic resonance spectroscopic/microscopic techniques or even two-photon microscopy/tomography and high-resolution ultrasound (Rallis and Koumantaki-Mathioudaki 2007).

The pathological response to the infection of Zebra fish with *M. marinum* was reported when this fish was inoculated with three different strains of *M. marinum* (Mma7, Mma11 and Mma20) isolated from different sources (van der Sar et al. 2004). Microscopic manifestations of fish infected with Mma20 encompassed extreme numbers of mycobacteria with loss of structural organization, severe necrosis, invasion of peritoneum and surrounding tissues with numerous inflammatory cells ending in severe peritonitis and early fish death. In case of Mma11 inoculation, organized granulomas were detected in liver, kidney pancreas, intestines and connective tissues. The infection with Mma7 strain was associated with the induction of fewer and less organized granulomas, which may have sometimes an outer layer of epithelial cells and a necrotic center (Rallis and Koumantaki-Mathioudaki 2007).

## 8. Diagnosis of *M. marinum* infections in fish

Mycobacterial cell wall resists the acid–alcohol decolorisation after being stained with carbolfuchsin. So Mycobacteria are revealed in fish tissue sections by staining with Ziehl–Neelsen acid-fast stain. The related actinomycetes including *Nocardia*, *Tsukamurella*, *Gordonia* and *Rhodococcus* are considered as partially acid-fast (Holt 1994), while *Legionella* spp. occasionally reveal some acid-fastness (Bentz et al. 2000). Acid-fast bacilli are frequently visible in piscine mycobacterial granulomas, although granulomas containing no visible acid-fast bacilli are reported in many experimentally infected species (Watral and Kent, 2007).

*Mycobacterium* could be effectively cultured on a variety of selective liquid and agar media (Rhodes et al. 2004), for example mycobactin is used to enhance the growth of slowly growing *Mycobacterium* on the standard media. Non-selective media are ineffective for slow growers but appropriate in the fast growers. It is necessary to differentiate between mycobacteria recovered from fishes, and fish-pathogenic mycobacteria. Aseptic isolation of *Mycobacterium* from internal organs confirms that the strains do not contain any contaminants (Rhodes et al. 2004).

The isolation of such bacteria from feces, external surfaces and whole viscera is questionable due to the presence of external contaminants (Rhodes et al. 2004). Tissue samples of fish are homogenized and then plated on appropriate culture media, including Middlebrook 7H10 or Lowenstein–Jensen media, to enhance *Mycobacterium* spp. growth. Being slow growing organisms, several *Mycobacterium* spp. necessities the maintenance of culture plates for a period ranging from 2 to 3 months before considering as negative.

Culture-based detection of *Mycobacterium* spp. from skin or gills of fish is complicated by the existence of background microbiota which compete *Mycobacterium* growth on the standard media. Because of its hydrophobicity, *Mycobacterium* is highly resistant to treatment with both acidic and basic chemicals and other compounds such as benzalkonium chloride and hypochlorite (Brooks et al. 1984; Rhodes et al. 2004). These substances have been utilized to isolate *Mycobacterium* from samples with a high microbial background, although they may also adversely affect the recovery of *Mycobacterium* (Brooks et al. 1984; Rhodes et al. 2004). Therefore, using the most appropriate decontamination procedures needs through evaluation for both minimum concentrations and exposure times.

Various molecular diagnostic tools have been established for detecting and identifying *Mycobacterium* spp. representing public health hazard. The small subunit, 16S rRNA gene is a popular target for accurate diagnosis due in part to the availability of *Mycobacterium* 16S gene sequences in the repositories as the GenBank and the Ribosomal Differentiation Database of Microorganisms. Identification of *M. marinum*, *M. fortuitum*, and *M. chelonae*, using restriction fragment length polymorphism analysis of the 16S rRNA gene (PCR-RFLP) was first explained in 1997 (Talaat et al. 1997). Moreover, DNA probe and gene sequencing have been used to identify *Mycobacterium* spp. up to the species level (Turenne et al. 2001; Chemlal and Portaels 2003).

Direct sequencing from fish tissues can be a useful option when the culture of Mycobacteria fails (Poort et al. 2006). Although numerous isolates of fish shared high homology in *Mycobacterium* genes, they require detection of multiple gene targets (Devulder et al. 2005). Recently, FRET (fluorescence resonance energy transfer) showed high specificity via melting curve analysis, which effectively discriminate and distinguish *M. marinum* from other mycobacteria (Salati et al. 2010). This diagnostic technique is summarized in a primary PCR with SYBR green (at a detection limit of 10^2^ *Mycobacterium* DNA copies) and then a secondary amplification using FRET in real-time PCR.

## 9. Management of *M. marinum* infections in fish

Complete control of piscine mycobacteriosis may need the destruction of all affected stocks and disinfecting the holding tanks and plumbing (Roberts 2001; Noga 2011). Ethanol, lysol and sodium chlorite have been reported efficiently capable of destroying *M. marinum* in aquaria, while potassium peroxymonosulfate is ineffective. Moreover, sodium hypochlorite is considered as a powerful sterilizing agent when the contact time was prolonged to more than 10 min (Mainous and Smith 2005). Nevertheless, researchers had stated that disinfections are not successful for controlling fish mycobacteriosis, due to the resistance of mycobacteria to common disinfectants (Jacobs et al. 2004). Furthermore, the Ag85A DNA vaccine protected striped bass for short term against *M. marinum*, but it could not provide long-term protection (Pasnik and Smith 2005).

## 10. Transmission to man

*M. marinum* penetrates the skin via minor traumata arising from resting the elbows on the border of the fish pond. Nowadays, the exposure of swimmers to mycobacteria was reduced due to the chlorination of water in the swimming pool (Ang et al. 2000). The growth of *M. marinum* increased in a Swedish dolphin atrium due to the decrease of water chlorination. It has coincided with numerous animals acquiring cutaneous and subcutaneous *M. marinum* infection. Additionally, infection may be acquired by handling shellfish or fish or following trauma attributable to infected foreign bodies for instance wood splints (Beecham et al. 1991).

However, the disease is not transmittable from person to person (Clark et al. 1990). Currently, most human infection cases happen with the exposure to aquaria (Aubry et al. 2002) as well as skin injuries occurring during the processing or preparations of seafood (Clark et al. 1990; Lawler 1994). The current practices of sanitary chlorination restrict largely the outbreaks of mycobacterial infections. The terms ‘fish tank granuloma’ and ‘fish handler's disease’ is often used due to the relation between home aquariums and the water-associated activities including swimming, fishing and boating (Ang et al. 2000).

*M. marinum* infections may have occupational hazards for pet shop workers. Many infections take place in fish fanciers who maintain the aquarium at home producing what is called fish fanciers ‘finger syndrome’ (Wheeler and Graham 1989). Some mycobacterial infection may take place through direct injury from fins of fish or bites, but most infections are acquired during aquarium handling, e.g. cleaning or water changing (Bhatty et al. 2000; Jernigan and Farr 2000). The incubation period of *M. marinum* infection in humans ranges between 3 weeks and 9 months (Jernigan and Farr 2000).

The evidence of zoonotic transmission of *M. marinum* had been formerly confirmed through studying the genetic linkages of isolates from both human and fish sources using pulsed field gel electrophoresis (Tsai et al. 2007; Slany et al. 2013) and amplified fragment length polymorphism (Doedens et al. 2008). However, in one human clinical case, *M. marinum* was inoculated through a fish spine injury (Tsai et al. 2007). Zoonotic infection had been ascribed to mycolactone toxin produced by mycobacteria (Chemlal et al. 2002). Although it is assumed that the local aquaculture industry is not the source of human infections in combination with the lack of records of human infections by some strains (*M. marinum* Eilaticum and *M. marinum* Cyprinum), but still only certain strains of *M. marinum* have zoonotic potential (Ucko and Colorni 2005).

## 11. Clinical presentation in human

Mycobacterial infections may be either painful or painless, but it may be life threatening (Wu et al. 2002). Infections with *M. marinum* may be hypothetically classified into four clinical categories (type I–type IV) to help guide therapeutic options (Bhatty et al. 2000). Type I *M. marinum* is commonly observed in immunocompetent patients and known as single or limited (1–3) lesions marked by superficial cutaneous infection, appearing in forms of crusted or ulcerated nodules or verrucous plaques. These single lesions develop within weeks or months of contact with infected fish (Aubry et al. 2002).

Type II *M. marinum* is numerous (more than 3) lesions with inflammatory nodules or in a sporotrichoid spreading pattern or with abscesses and granulomas in an immune-suppressed patient. This ‘sporotrichoid’ infection starts with distal inoculation and can progress to nodular lymphangitis (Bartralot et al. 2005). Type III *M. marinum* appears as deep infections associated with or without skin infection signs including arthritis, tenosynovitis, osteomyelitis and/or bursitis. Type IV *M. marinum* is disseminated infection with lung disease and other systemic manifestations. Bacteraemia is very rare but may occur in severely immuonocompromised patients (Bhatty et al. 2000). Other sites of *M. marinum* infection were also reported. Severe intranasal lesions resulted also from *M. marinum* infection (Asakura et al. 2016), while the disseminated infection comprised the skin, the viscera and the lung (Petrini 2006) and laryngeal lesions (Gould et al. 1968).

## 12. Treatment of *M. marinum* infections in man

The proper treatment of *M. marinum* infection is practically required for rapid recovery in man, avoiding progress to deeper tissues. Insufficient data about efficacy of many treatment regimens (Gluckman 1995) render the treatment fairly difficult. Moreover, *M. marinum* is a naturally multi-drug resistant organism, and the treatment is primarily dependent on the investigators and their personal experiences (Edelstein 1994). Monotherapy is usually effective in the infection of skin and soft tissue, but this is not sufficient in deeper infections. In the superficial cutaneous infection, monotherapy including clarithromycin, trimethoprim and ciprofloxacin was reportedly considered as an effective treatment, while in case of deeper infections combination therapy of two drugs may be more effective.

In case of a sporotrichoid distribution pattern, a combination of two drugs, namely ethambutol and rifampicin is recommended. Streptomycin, isoniazid and pyrazinamide should be excluded from treatment regimens because *M. marinum* is more resistant. Treatment of infected deeper structure takes longer periods and the acquired resistance has not been recorded in *M. marinum* while clinical awareness is very important to initiate the diagnosis and the treatment (Aubry et al. 2002). Various therapeutic alternatives such as electrodessication, therapy with X-ray, cryotherapy, local hyperthermic therapy and photodynamic therapy have been recorded and recommended (Rallis and Koumantaki-Mathioudaki 2007). Although phage therapy using the mycobacteriophage D29 proved effective in the treatment of Buruli Ulcer (BU) caused by *M. ulcerans* in the murine footpad model (Trigo et al. 2013), there is no bacteriophage specific for controlling *M. marinum* infections.

## 13. Conclusions

Piscine mycobacteriosis arising from *M. marinum* infection can have adverse effects on the fish of fresh water, marine and brackish water, constituting zoonotic implications. The data on the pathogenesis and virulence factors of *M. marinum* are still scarce and questionable. Further studies are required to trace the immune defense responses against *M. marinum* infection in fish. Prophylaxis and vaccine development are extremely important to protect different aquaria from *M. marinum* infection. The advent of molecular diagnostic tools and advanced epidemiological studies on fish mycobacteriosis can enhance our understanding of the strategic plans designed for controlling such disease. Due to its communicable public health hazard, the disease should be cautiously avoided by voiding swimming in fish pools and wearing gloves during handling and processing of fish. Future studies should also evaluate the application of phage therapy in the fish aquarium to prevent and control *M. marinum* infection.
